# Thromboembolic events in cancer patients on active treatment with cisplatin-based chemotherapy: another look!

**DOI:** 10.1186/s12959-018-0161-9

**Published:** 2018-03-01

**Authors:** Hikmat Abdel-Razeq, Asem Mansour, Hazem Abdulelah, Anas Al-Shwayat, Mohammad Makoseh, Mohammad Ibrahim, Mahmoud Abunasser, Dalia Rimawi, Abeer Al-Rabaiah, Rozan Alfar, Alaa’ Abufara, Alaa Ibrahim, Anas Bawaliz, Yousef Ismael

**Affiliations:** 1 Department of Internal Medicine, King Hussein Cancer Center, 202 Queen Rania Al-Abdulla St., P.O. Box 1269, Amman, 11941 Jordan; 2 Radiology, King Hussein Cancer Center, Amman, Jordan; 3 Scientific and Reseaerch Office, King Hussein Cancer Center, Amman, Jordan; 4 Pharmacy, King Hussein Cancer Center, Amman, Jordan; 5 Radiation Oncology, King Hussein Cancer Center, Amman, Jordan

**Keywords:** Cisplatin, Chemotherapy, Thrombosis, Cancer

## Abstract

**Background:**

The risk of thromboembolic events is higher among cancer patients, especially in patients undergoing chemotherapy. Cisplatin-based regimens claim to be associated with a very high thromboembolic rate. In this study, we report on our own experience with thrombosis among patients on active cisplatin-based chemotherapy.

**Methods:**

Medical records and hospital databases were searched for all the patients treated with any cisplatin-based regimen for any kind of cancer. Thrombosis was considered cisplatin-related if diagnosed any time after the first dose and up to 4 weeks after the last. The Khorana risk assessment model was performed in all cases.

**Results:**

A total of 1677 patients (65.5% males, median age: 50 years) treated with cisplatin-based regimens were identified. Head and neck (22.9%), lung (22.2%), lymphoma and gastric (11.4% each) were the most common primary tumors. Thromboembolic events were reported in 110 (6.6%); the highest was in patients with gastric cancer (20.9%) and the lowest in patients with head and neck cancers (2.3%) and lymphoma (1.6%). Thrombosis included deep vein thrombosis (DVT) in 69 (62.7%), pulmonary embolism (PE) in 18 (16.9%) and arterial thrombosis in 17 (15.6%). A majority (51.1%) of the patients had stage IV disease and only 16% had stage I or II.

In a multivariate analysis, significantly higher rates of thrombosis were associated with gastric as the primary tumor, advanced-stage disease, female sex but not age, and the Khorana risk score or type of cisplatin regimen. While the presence of CVC was significantly associated with the risk of thrombosis (*p* < 0.0001) in the univariate analysis, and such significance was lost in the multivariate analysis (odds ratio, 1.098; 95%CI, 0.603–1.999, *p* = 0.7599).

**Conclusions:**

Thromboembolic events in cancer patients on active cisplatin-based chemotherapy were commonly encountered. Gastric cancer, regardless of other clinical variables, was associated with the highest risk.

## Background

Venous thromboembolism (VTE) and, to a lesser extent, arterial thrombosis are common complications encountered in patients with cancer during the course of their treatment and follow-up [1, 2].

Much of this high risk is attributed to the cancer itself or its therapy. However, patient-related factors such as age, performance status, body mass index and underlying comorbidities are also important factors [3, 4].

Thromboembolic events are one of the leading causes of death in patients with cancer [5]. Many studies show that the survival of cancer patients with thrombosis is significantly lower than those without [6–8].

Cisplatin is an old chemotherapeutic drug that was licensed in 1978 and is now listed on the World Health Organization’s list of essential medicines. It is widely used, alone or in combination, to treat a number of cancers, including testicular, ovarian, cervical, bladder, head and neck, lung, esophageal and gastric cancers [9, 10].

Cisplatin is well known for its vascular and thrombotic complications, including both venous and arterial thrombosis [11–13]. In one study, researchers at Memorial Sloan-Kettering Cancer Center (MSKCC) and Michigan State University reported a thrombosis rate of 18.1% among 932 patients treated with cisplatin-based regimens for various kinds of cancers. All had their thromboembolic events while on active cisplatin therapy or within 4 weeks of the last dose. Deep vein thrombosis (DVT) and/or pulmonary embolism (PE) accounted for almost 90% of the events [14]. More recently, a higher risk of VTE [crude relative risk of 2.8 (95% CI, 1.4–4.2)] was also reported in a group of 200 patients with various malignancies undergoing treatment with cisplatin-based chemotherapy compared to 200 others who received non-cisplatin-based chemotherapy [15]. A meta-analysis that involved 8216 patients treated with different chemotherapy regimens for various advanced solid tumors from 38 randomized controlled trials was recently reported. Patients receiving cisplatin-based chemotherapy had a significantly increased risk of VTEs (RR, 1.67; 95% CI, 1.25 to 2.23; *P* = 0.01) [16].

Given the variation in the reported thromboembolic rates among such patients, this study highlights the observed thrombosis rate in real daily clinical practice. Factors that help predict the occurrence of thrombosis were also studied. Following this analysis, it was hypothesized that particular group(s) of patients with specific clinical features and particular primary tumors treated with cisplatin-based regimen could be identified as high risk for VTE to justify prophylaxis, even in the ambulatory setting. The results of this study could help design clinical trials addressing preventive measures for a subgroup of patients with the highest risk.

## Methods

Medical records and the hospital database were searched for patients treated with any cisplatin-based regimen for any kind of cancer. All adult patients (≥ 18 years old) that were treated between January 2007 and December 2015 and had at least 4 weeks of follow-up after their last cisplatin dose were included.

The patients’ medical records and imaging reports were searched for a diagnosis of venous or arterial thrombosis. To avoid any missing events, we also searched the pharmacy database for any anticoagulant therapy for all the patients who received cisplatin-based chemotherapy during this period.

The presence of central venous catheter (CVC) and other thrombotic risk factors used to calculate the Khorana risk score were collected, and such risk factors included hemoglobin level, platelet and WBC counts, primary cancer site, disease stage, and body mass index (BMI) [17]. Patients were then grouped into three risk categories, including high, intermediate and low risk, as shown in Table 1. Thrombosis was considered cisplatin-related if it was diagnosed any time after the first dose and up to 4 weeks after the last. All DVT was diagnosed by Doppler ultrasound, while all the PE were diagnosed by CT angiogram. This study was approved by our institutional review board.

**Table 1:** Khorana Risk Assessment Model

Patient characteristic	Risk Score
1. Site of cancer	
▪ Very high risk (stomach, pancreas)	2
▪ High risk (Lung, Lymphoma, Gynecologic, bladder, testicular)	1
2. Prechemotherapy platelet count 350 × 10^9^/L or more	1
3. Hemoglobin level less than 100 g/L or use of red cell growth factors	1
4. Prechemotherapy leukocyte count more than 11 × 10^9^ /L	1
5. BMI: 35 kg/m^2^ or more	1

Three Risk Groups:

▪ Low Risk 0

▪ Intermediate Risk 1–2

▪ High Risk ≥3

### Statistical analysis

The primary objective of this study was to determine the overall incidence and characteristics of thrombosis in adult patients receiving cisplatin-based chemotherapy with or without radiation therapy. The secondary objectives were to analyze the importance of the patients’ tumor and treatment characteristics in predicting the occurrence of thrombosis.

The association between such variables and the development of thromboembolic events during the defined treatment period was evaluated using the *X*^*2*^ test for categorical variables and the Wilcoxon rank sum test for continuous variables. The variables found to be significant (*p* < 0.05) by the univariate analysis were subsequently entered into a multivariate logistic regression model. Following this analysis, it was hypothesized that particular group(s) of patients with specific clinical features and particular primary tumors treated with cisplatin-based regimen could be identified as high risk for VTE to justify prophylaxis, even in the ambulatory setting.

## Results

During the study period, 1677 patients received at least one cycle of cisplatin-based regimen and were included in this study. The median age was 50 years (range: 18–83 years), and 1099 (65.5%) were male. The most common primary tumors encountered were head and neck (22.9%), lung (22.2%), lymphoma (11.4%), gastric (11.4%), and testicular (8.4%). A majority of the patients had advanced-stage disease at the time of chemotherapy administration, and 858 (51.2%) patients were stage IV and 487 (29.0%) patients had stage III disease. Chemotherapy was delivered through a central venous catheter (CVC) in 303 (18.1%) patients as shown in Table 2. None of the patients had any form of thromboprophylaxis while in ambulatory settings.

**Table 2:** Patients Characteristics (*n* = 1677)

Characteristic	No. of Patients		Percentage
Age (Years)			
Median		50	
Range		18–83	
Sex			
Male	1099		65.5
Females	578		34.5
Primary Tumor			
Head and Neck	384		22.9
Lung	373		22.2
Gastric	191		11.4
Lymphoma	191		11.4
Cervical	121		7.2
Testicular	104		6.2
Bladder	77		4.6
Sarcoma	45		2.7
Esophageal	33		2.0
Others	158		9.4
Khorana Risk Score			
Low	350		20.9
Intermediate	1075		64.1
High	252		15.0
Central Venous Catheter			
Present	303		18.1%
Absent	1374		81.9%
Disease Stage			
I	56		3.3
II	213		12.7
III	487		29.0
IV	857		51.1
Unstageable/Unknown	64		3.8

Thromboembolic events were reported in 110 (6.6%) patients; 96 (5.7%) were venous thrombosis in the form of DVT and/or PE, and 14 (0.83%) had arterial thrombosis. The thrombosis rate was highest among patients with gastric cancer; it was reported in 40 (20.9%) of 191 patients compared to 70 (4.7%) of 1486 patients with other tumor types, *p* < 0.0001. The thromboembolic rates were particularly low among the patients with lymphoma (1.6%), head and neck (2.3%) and testicular cancers (2.8%).

The thrombosis rate was also studied in relation to the Khorana risk score. Patients with a high-risk score had higher rates of thromboembolic events, which were reported in 33 (13.1%) of 252 such patients compared to 77 (5.4%) of all the other patients with an intermediate or low-risk score, *p* < 0.0001, as shown in Table 3.

**Table 3:** Thromboembolic events for the whole group

	Whole Group (1677)		Patients with thrombosis (110)		
	Number	%	Number	%	*p*-value
Sex					
Male	1099	65.5	58	5.3	0.003
Female	578	34.5	52	9.0	
Age (Years)					
Missed	1				0.6445
≤ 60	1276	76.1	81	6.3	
> 60	400	23.9	28	7.0	
Primary Tumor					
Head and Neck	384	22.9	9	2.3	<0.0001
Lung	373	22.2	25	6.7	
Gastric	191	11.4	40	20.9	
Lymphoma	191	11.4	3	1.6	
Testicular	104	6.2	4	3.8	
Cervical	121	7.2	7	5.8	
Bladder	77	4.6	3	3.9	
Sarcoma	45	2.7	3	6.7	
Esophageal	33	2.0	6	18.2	
Others	158	9.4	10	6.3	
Khorana Risk Score					
Low risk	350	20.9	16	4.6	<0.0001
Intermediate risk	1075	64.1	61	5.7	
High risk	252	15.0	33	13.1	
Disease Stage					
I	56	3.3	3	5.4	0.0038
II	213	12.7	8	3.8	
III	487	29	20	4.1	
IV	857	51.1	76	8.9	
Unstageable/Unknown	64	3.8	3	4.7	

We also studied the effect of a combination chemotherapy regimen on the incidence of thrombosis. This rate was highest (30.0%) among a small group of 30 patients treated with ECF (epirubicin, cisplatin and fluorouracil (5-FU)); all of these patients had gastric cancer. The rate of thrombosis was 12.7% in the 245 treated with cisplatin, docetaxel and 5-FU, was only 2.1% among the 145 patients treated with cisplatin and etoposide and was 4.3% among the 116 patients treated with the BEP (bleomycin, etoposide and cisplatin) regimen or cisplatin and radiation therapy. Table 4 shows the chemotherapy regimens and corresponding thromboembolic events.

**Table 4:** Thromboembolic events according to chemotherapy regimen

Chemotherapy Regimen	Number of Patients	Thromboembolic Events	
		(n)	%
Cisplatin-XRT	299	13	4.3
Cisplatin-Docetaxel-5FU	245	31	12.7
Cisplatin-Docetaxel	194	13	6.7
DHAP	180	3	1.7
Cisplatin-Etoposide	145	3	2.1
Cisplatin-5FU	120	9	7.5
BEP	116	5	4.3
Cisplatin-Gemcitabine	95	9	9.5
Cisplatin-Doxorubicin	42	3	7.1
Cisplatin-Etoposide-XRT	38	4	10.5
ECF	30	9	30.0
Cisplatin-Pemetrexed	19	3	15.8
Others	154	5	3.2

*5-FU* 5-Flurouracil, *DHAP* Dexamethasone, High-dose

Ara-C and Cisplatin; *BEP* Bleomycin, Etoposide and Cisplatin, *ECF* Epirubicin, Cisplatin and 5-Flurouracil, *XRT* Radiation therapy

To further address the effect of the chosen combination chemotherapy regimen in relation to a particular disease, we studied the three most commonly utilized regimens, including Cisplatin-Radiation (299 patients), Cisplatin-Docetaxel-5FU (245 patients) and Cisplatin-Docetaxel (193 patients). While none of the 95 patients with head and neck cancers treated with (Cisplatin-Docetaxel-5FU) had any thromboembolic events, 28 (20.6%) of the 136 patients with gastric cancer and 3 (42.9%) of the 7 patients with esophageal cancers treated with the same regimen had thrombosis, *p* < 0.0001. Further details are shown in Table 5.

**Table 5:** Thromboembolic events according to Cisplatin-based chemotherapy regimen

Chemotherapy Regimen	Patients		Thromboembolic Events		*p*-value
	(n)	(%)	(n)	(%)	
Cisplatin-Docetaxel-5FU					
Head and Neck	95	38.8	0	0	<0.0001
Gastric	136	55.5	28	20.6	
Esophageal	7	2.9	3	42.9	
Others	7	2.9	0	0	
Total	245		31	12.7	
Cisplatin-XRT					
Cervical	101	33.8	7	6.9	0.2166
Head and Neck	184	61.5	5	2.7	
Others	14	4.7	1	7.1	
Total	299		13	4.3	
Cisplatin-Docetaxel					
Lung	181	93.3	12	6.6	0.9999
Others	13	6.7	1	7.7	
Total	194		13	6.7	

*5FU* 5-Flurouracil, *XRT* Radiation therapy

We further analyzed the 191 gastric patients and studied the rate of thrombosis in relation to many other variables, including the chosen combination chemotherapy, the disease stage, the Khorana risk score and age. None of those clinical variables had a significant impact on the rates of thromboembolic events, as shown in Table 6.

**Table 6:** Thromboembolic events in patients with Gastric cancer

Clinical Variables	Number of Patients	Thromboembolic Events		*p*-Value
		(n)	(%)	
Chemotherapy Regimen				
ECF	26	8	30.8	0.5982
Cisplatin-Docetaxel-5FU	137	28	20.4	
Cisplatin-5FU	20	4	20	
Others	8	0	0	
Disease Stage				
Early Stage	35	11	31.4	0.0916
Metastatic	156	29	18.5	
Khorana Risk Score				
Low^a^	1	1		0.5331
Intermediate	101	19	18.8	
High	89	20	22.5	
Age (years)				
Missed	1			0.8647
< 50	90	18	18.5	
≥ 50	100	21	23.5	

*ECF* Epirubicin, Cisplatin and 5-Flurouracil, *5-FU* 5-Flurouracil

^a^ Only one patient and had thrombosis

To further address the association of the baseline and treatment variables with the development of thrombosis, a univariate analysis was conducted. Sex, the Khorana risk score, the presence of central venous catheter (CVC), and the primary tumor site and stage were all associated with a significantly higher rate of thrombosis as shown in Table 7.

**Table 7:** Univariate Analysis for thromboembolic events

Factors		Number of Patients	Thromboembolic Events 110 (6.6%)	*P*-value
Age	≤ 60	1276 (76.1%)	81 (6.3%)	0.6445
	> 60	401 (23.9%)	29 (7.2%)	
Gender	Female	578 (34.5%)	52(9.0%)	0.003
	Male	1099 (65.5%)	58 (5.3%)	
Khorana risk	High	252 (15.0%)	33 (13.1%)	<0.0001
	Others	1423(85.0%)	77 (5.4%)	
Central venous catheter	No	1374 (81.9%)	70 (5.1%)	<0.0001
	Yes	303 (18.1%)	40 (13.2%)	
Primary Tumor	Gastric	191 (11.4%)	40 (20.9%)	<0.0001
	Other	1486 (88.6%)	70 (4.7%)	
Stage	IV	857 (51.1%)	76 (8.9%)	<0.0001
	Early stage	756 (45.1%)	31 (4.1%)	

The significant variables in the univariate analysis were subsequently entered into a multivariate logistic regression model. In the multivariate analysis, only sex (odds ratio, 1.732; 95% CI, 1.152–2.605, *p* = 0.0083), gastric cancer as the primary site (odds ratio, 3.377; 95% CI, 1.759–6.483, *p* = 0.0003) and disease stage (odds ratio, 1.665; 95% CI, 1.054–2.63, *p* = 0.0289) were significantly associated with thromboembolic events as shown in Table 8. In addition, the presence of CVC was significantly associated with risk of thrombosis (*p* < 0.0001) in the univariate analysis, and this significance was lost in the multivariate analysis (odds ratio, 1.098; 95%CI, 0.603–1.999, *p* = 0.7599).

**Table 8:** Multivariate Analysis for thromboembolic events

Factor	Odds Ratio	95% Confidence Limits	*P*-value
Primary Tumor (Gastric vs. Others)	3.377	1.759–6.483	0.0003
Gender (Female vs. Male)	1.732	1.152–2.605	0.0083
Khorana risk group (High vs. Others)	1.387	0.842–2.285	0.1992
Central Venous Catheter (Presence vs. Absence)	1.098	0.603–1.999	0.7599
Stage (IV vs. Early stage)	1.665	1.054–2.63	0.0289

## Discussion

The association of cisplatin with thrombosis is well-known. However, its pathogenesis remains unclear. Endothelial cell damage, as revealed by the increased plasma levels of the Von Willebrand factor during chemotherapy, is believed to be a major contributing factor [18]. Platelet activation and the up-regulation of prothrombotic factors are also implicated in cisplatin-associated thrombosis [19–21].

In addition to venous thrombosis, arterial thrombosis is also well-described. In one study, 25 cases of myocardial infarction (MI) and cerebrovascular accidents (CVA) were reported among a group of young patients treated with cisplatin-based regimens for testicular cancer. None of these patients had known risk factors and none had atherosclerotic features [22].

Given the high recurrence rates [23], poor quality of life, and worse overall survival associated with thrombosis in cancer patients [6–8, 24], antithrombotic prophylaxis is widely practiced. Much of the emphasis is given to patients admitted for medical illnesses or surgical procedures. Such practice was endorsed by many international clinical practice guidelines, including the American Society of Clinical Oncology (ASCO) and the National Comprehensive Cancer Network (NCCN) [25, 26].

However, many recent studies addressing thrombosis in cancer patients show that a significant portion of these patients had thrombosis while in an ambulatory setting and were never admitted at the time or just prior to their thrombotic events [27]. Many factors contributed to these findings, and routine oncology practice has recently shifted away from the inpatient setting to the ambulatory one. Additionally, we tend to offer chemotherapy to much older and sicker patients. Routine thrombotic prophylaxis for such ambulatory cancer patients is not endorsed by any of the published guidelines.

Several efforts have been made to address the issue of prophylaxis among ambulatory cancer patients on active chemotherapy. Khorana, et al. suggested a risk assessment model that assigns these patients into three risk levels, including high, intermediate, and low [17]. Despite its simplicity and the availability of the data needed to calculate the risk of VTE, this model failed to gain popularity in clinical practice, and several studies show that it can be only applied to a small portion of such ambulatory patients [28]. Additionally, although the Khorana score, detailed in Table 1, considered gastric cancer as a high-risk type (score of 2), it did not consider the type of chemotherapy offered as a risk category.

Enrolling high-risk patients in clinical trials to test the value of thromboembolic prophylaxis in a wide range of cancer patients was another promising approach. However, due to a higher risk of bleeding and despite the associated benefit in lowering the thrombosis rate, this approach also failed to show a significant overall clinical benefit [29, 30].

A third approach was offering VTE prophylaxis to a particular high-risk group of patients with a specific diagnosis, such as advanced pancreatic cancer, undergoing active chemotherapy. The benefit of this approach was also offset by the high rate of bleeding [31, 32]. Another approach, similar to the one under discussion in this study, is to link a specific kind of chemotherapy and thrombosis risk. Cisplatin, as discussed earlier, is a good example.

Our thrombosis rate was lower than the 18% reported by the MSKCC group [14]. Many factors could have contributed to this lower rate, including a lower risk-patient population enrolled and different diagnostic methods. However, clinically important observations were noted and deserve discussion. In our study, we identified a specific subgroup of cancer patients treated with cisplatin-based chemotherapy with a real high risk for thrombosis. Patients with gastric cancer had a significantly higher rate of thrombosis, which was 20.9% compared to 4.7% among other patients receiving a similar cisplatin-based chemotherapy regimen. This high rate of thrombosis among patients with gastric cancer was high regardless of their age, stage, Khorana risk score, or the combination chemotherapy regimen used as shown in Table 6.

Given this relatively high rate, we proposed here that such patients could be selected into a randomized clinical trial to test the value of thrombotic prophylaxis among them, and most of them were usually treated with chemotherapy in the ambulatory setting without prophylaxis.

Thromboembolic prophylaxis in particular disease entities undergoing specific combinations of chemotherapy is routinely practiced in diseases, such as multiple myeloma (MM). We now have strong evidence and clear guidelines to offer antithrombotic prophylaxis when these patients are treated with immune modulators (thalidomide and lenalidomide) when combined with dexamethasone [25, 26].

We hope that future clinical research will lead to clear guidelines recommending antithrombotic prophylaxis in high-risk cancer patients, such as those with gastric cancer treated with a cisplatin-based regimen, even when done in ambulatory settings similar to what we routinely do with MM patients.

## Conclusions

Thromboembolic events among cancer patients on active cisplatin-based chemotherapy are relatively common. The highest thrombosis rates were encountered in patients with gastric cancer regardless of other clinical variables. Prospective randomized trials are needed to study the value of VTE prophylaxis in such high-risk patients.

## Abbreviations

5-FU: Fluorouracil
ASCO: American Society of Clinical Oncology
BEP: Bleomycin, Etoposide and Cisplatin
BMI: Body mass index
CVA: Cerebrovascular accidents
CVC: Central venous catheter
DHAP: Dexamethasone, High-dose Ara-C and Cisplatin
DVT: Deep vein thrombosis
ECF: Epirubicin, Cisplatin and Fluorouracil
MI: Myocardial infarction
MM: Multiple myeloma
NCCN: National Comprehensive Cancer Network
PE: Pulmonary embolism
VTE: Venous thromboembolism
XRT: Radiation therapy
